# The Effect of Tissue Stromal Vascular Fraction as Compared to Cellular Stromal Vascular Fraction to Treat Anal Sphincter Incontinence

**DOI:** 10.3390/bioengineering10010032

**Published:** 2022-12-26

**Authors:** Wenbin Chen, Zijian He, Shuyu Li, Zixin Wu, Jin Tan, Weifeng Yang, Guanwei Li, Xiaoling Pan, Yuying Liu, Feng-Juan Lyu, Wanglin Li

**Affiliations:** 1 Department of Colorectal and Anal Surgery, The Second Affiliated Hospital, School of Medicine, South China University of Technology, Guangzhou 510641, China; 2 School of Biology and Biological Engineering, South China University of Technology, Guangzhou 510641, China; 3 The Sixth Affiliated Hospital, School of Medicine, South China University of Technology, Guangzhou 510641, China

**Keywords:** cVSF, tSVF, anal sphincter incontinence

## Abstract

Background: The long-term prognosis of current treatments for anal sphincter incontinence (ASI) is poor. Here, we explored the efficacy of tissue adipose stromal vascular fraction SVF (tSVF) on ASI and compared it to that of cellular SVF (cSVF). We then investigated possible mechanisms. Methods: Rat cSVF and tSVF were isolated and labeled with DIL. One day after modeling, three groups received phosphate-buffered saline (PBS), cSVF, tSVF, respectively. The control group received nil modeling nor any treatments. The effect was assessed by function test for anal pressure and electromyography, and staining for fiber content, proliferation and differentiation at day 5 and day 10. Results: cSVF injection resulted in faster healing than tSVF. The cSVF group showed significant improvement on anal pressure on day 10. For the electromyography test, cSVF showed significant improvement for the frequencies on day 10, and for the peak values on both time points, while tSVF showed significant improvement for the peak values on day 10. The two SVF both alleviated fibrosis. Immunofluorescence tracing identified differentiation of some injected cells towards myosatellite cells and smooth muscle cells in both SVF groups. For all the tests, the tSVF group tends to have similar or lower effects than the cSVF group with no significant difference. Conclusion: cSVF and tSVF are both safe and effective in treating ASI, while the effect of cSVF is slighter higher than tSVF.

## 1. Introduction

Anal sphincter incontinence (ASI) is fecal incontinence caused by structural or functional dysfunction of the anal sphincter [1]. Although ASI does not usually result in death, it is often associated with a reduced quality of life and leads to psychological problems such as anxiety [2]. Obstetric trauma, surgical injuries, etc. are major causes of ASI [3,4]. Current treatments for ASI include conservative treatments such as dietary therapy and biofeedback therapy, as well as surgical treatments including anal sphincteroplasty and artificial anal sphincters [5,6]. However, the efficacy of these treatments is not satisfactory [7,8,9,10], as the effect of the treatment is not significant in the long term.

Stem cells are a powerful tool for regenerative medicine [11,12,13,14]. Adipose grafting was first used for soft tissue defect reconstruction in 1893 [15]. In 2001, Zuk et al. [16] obtained stromal vascular fraction (SVF) after processing and found a large number of multipotent cells that could differentiate into adipocytes, chondrocytes, myoblasts, and osteoblasts. Later, these cells were named adipogenic stem cells (ADSC). SVF is a heterogeneous population of cells that includes ADSC, as well as other components such as endothelial cells, lymphocytes, and hematopoietic lineage cells [17,18,19]. Some clinical trials have applied SVF to soft tissue reconstruction [20], lung repair [21], cartilage repair [22], and cardiac repair [23] with encouraging results.

The current concept of SVF contains two different types: cellular SVF (cSVF) obtained by enzymatic digestion of fat, and tissue SVF (tSVF) obtained by mechanical treatment of adipose tissue [24]. cSVF contains ADSC, pericytes, endothelial cells, macrophages and other cellular components [19,25], while tissue stromal vascular fraction (tSVF) also retains extracellular matrix (ECM) as a temporary scaffold [26] and paracrine factors [27], in addition to the factors in cSVF, which can provide structural and functional support [28] to the cellular parts and synergistically play a therapeutic role.

CSVF has a wide range of applications in medicine, including breast augmentation [29], post-radiotherapy injury in breast cancer patients [30], diabetic foot ulcers [31], urethral stricture [32], Crohn’s disease [33], etc. TSVF has also seen an increase in research in recent years [34]. Although cSVF has a longer history of utilization in medicine, cell–cell communication and ECM are lost in the preparation process [35], while the enzymatic digestion method is costly and time-consuming [36]. Moreover, the US and European Food and Drug Administrations do not allow the use of enzymatic digested bioproducts in the clinic [37]. In contrast, mechanical separation is a simple, inexpensive, rapid, and safer process than enzymatic digestion [38] which makes tSVF a more suitable stem cell source over cSVF in clinical development.

Currently, there is no study investigating the effect of tSVF on anal repair and it remains unclear which SVF is more advantageous in promoting anal sphincter repair. Here, we aim to comprehensively evaluate and compare the reparative effects of tSVF and cSVF on anal sphincter injury through a rat anal sphincter injury model. The effect was assessed by gross healing of the injury site, tissue staining for histological changes, anal pressure, and electromyography for functional tests, with the aim to compare the advantages and disadvantages of these two SVF in anal repair. We also examined the proliferation and differentiation of SVF to target cells to understand their fate in vivo. In total, our study provides more potential on the application of SVF, and suggests possible cell tools for anal sphincter repair.

## 2. Materials and Methods

### 2.1. Preparation of cSVF and tSVF

After the execution of SD rats using excess CO_2_, adipose tissue was collected and rinsed for 2 times with phosphate-buffered saline (PBS). Visible connective tissue and blood vessels were removed using ophthalmic scissors, then the tissue was cut into a paste. For cSVF preparation, half of the tissue was clipped and transferred to a 50 mL centrifuge tube, then an equal amount of 0.075% type I collagenase was added and placed in a water bath shaker at 37 °C for 30 minor digestion. After the completion of the digestion, an equal amount of low sugar complete DMEM medium was added to neutralize the enzyme. The neutralized mixture was filtered through a 70 μm filter, and centrifuged at 1500× *g* rpm for 5 min. The bottom sediment after centrifugation was resuspended in 1 mL of DMEM medium and incubated in 4 mL of erythrocyte lysate for 8 min. Then, it was centrifuged at 1500× *g* rpm for 5 min and resuspended with 1 mL of medium for cell counting. For tSVF preparation, the other half of the paste-like adipose tissue was transferred into a 20 mL syringe connected with a SVF converter with pore size 2.5 mm, and repeatedly passed the tissue through the converter at a uniform speed for 30 times. Then, it was replaced by another SVF converter with 1mm pore size and continued to repeatedly pass the tissue through the converter at a uniform speed for 20 times. The treated mixture was centrifuged at 2000× *g* rpm for 3 min and the second layer of tSVF tissue after centrifugation was collected and subsequently labeled with DIL.

#### DIL Labeling of cSVF and tSVF

For the label of cSVF, 2×10^7^ cSVF, cells were resuspended with 1 mL of culture medium and incubated with 2 μL DIL stock solution (1 μg/μL) for 5 min at room temperature in the dark, and then incubated further for 15 min in a refrigerator at 4oC. For the label of tSVF, 0.2 mL tSVF tissue was resuspended in 5 μm DIL stock solution in the same way. To check the labeling effect, 10 μL of labeled cSVF and tSVF was incubated with subadditive blue and observe under a microscope (Zeiss, Axio Observer3, Jena, Germany) to visualize cell fluorescence.

### 2.2. Animal Model 

The study was approved by the Experimental Animal Ethics Committee of South China University of Technology. The anal sphincter incontinence model was established by the excision of 25% of the ventral anal sphincter complex [39]. Thirty-two 6–8 week female Sprague Dawley (SD) rats (190–220 g) were randomly divided into control (*n* = 8), PBS-treated (*n* = 8), cSVF-treated (*n* = 8) and tSVF-treated (*n* = 8) groups. The control group was not given any intervention. The rats were first anesthetized by intraperitoneal injection with 0.2% sodium pentobarbital (0.3 mL/100 g). The surgical area was shaved and disinfected, and the resection area was marked. The ventral 25% of the anal sphincter complex was excised for about 1 cm × 0.5 cm. Buprenorphine was given for analgesia for 2 days after surgery with standard post-operative care. The PBS treatment group, the cSVF group, and the tSVF group received the injection of 0.2 mL of PBS, 2 × 10^7^ cSVF cells in 0.2 mL PBS, or 0.2 mL of tSVF tissue into both sides of the injury site at 1 day after modeling, respectively. After 5 days of treatment, half of the animals received function measurement before execution to harvest the tissue. The other half of the rats were tested for anal function at day 10 and harvested for staining.

### 2.3. Functional Assay

The anal pressure test was performed as in the previous study [40], i.e., after the anesthetized rats were secured with cellophane glue, the pressure transducer was connected after emptying the air bubbles in the balloon catheter. Then, the bioinformatics acquisition system hardware and software (RM6240E Bioinformatics Acquisition System, Chengdu, China) were connected at 30 min in advance, and the lubricated balloon was inserted into the rat’s anus to the extent that the balloon just exceeded the edge of the anus by approximately 4 mm. Next, 0.5 mL of saline was injected into the balloon prior to recording. The waveform was recorded for about 15 min. The rat anal sphincter EMG test was performed with three 30G EMG needles connected to the bioinformatics acquisition system through electrode clips at the proximal end and at the distal end at the subcutaneous position of the rat’s front paw and at the 3 and 9 points of the anal sphincter, respectively, and recorded for 15 min after the appearance of regular EMG waveforms.

### 2.4. Masson Trichrome Stain

After the functional test, the animals were executed with excess CO_2_. Tissue specimens were collected from the anal region, the external skin was peeled off, the specimens were soaked in 10% formalin, and then the specimens were embedded using paraffin and cut into 6um sections. The Masson trichrome stain was performed according to the manufacturer’s instructions (Guge Biology, G1006, Wuhan, China).

### 2.5. Immunofluorescence Staining

The fixed specimens were sectioned, dehydrated, and antigenically repaired with EDTA antigen repair buffer (pH = 9.0), then the tissue was uniformly incubated with 10% bovine serum albumin (BSA) in a humidified chamber at room temperature for 30 min. The sections were then incubated with the antibodies against proliferating cell nuclear antigen (PCNA, PTG,10205-2-A, Wuhan, China) for proliferation, against α-smooth muscle actin (α-SMA, PTG,14395-1-A, Wuhan, China) for smooth muscle differentiation, and against myogenic differentiation factor D (MyoD, PTG,18943-1-A, Wuhan, China) for myosatellite differentiation. After incubation overnight at 4 °C, the tissue was washed three times using PBS solution (pH = 7.4), covered with secondary antibodies and incubated for 50 min with protection from light, followed by dropwise incubation with 40,6-diamidino-2-phenylindole (DAPI) stain for 10 min and sealed with anti-fluorescence quenching blocker. Staining was observed and data collected using an inverted fluorescence microscope (Nikon, Eclipse Ti-SR, Tokyo, Japan).

### 2.6. Data Analysis

All continuous variables are expressed as mean ± standard deviation. Immunofluorescence intensity and fiber quantification after Masson staining were analyzed using Image J software. For the fluorescence signal quantification, the fluorescent images were converted to grayscale, inverted and subjected to optical density correction and threshold adjustment until all target cells were selected. Then, the average optical density of the area of positive measurement was exported and compared between groups. The quantitation of Masson trichrome staining was conducted in a similar way by adjusting the threshold until all fibers were selected. Then, the percentage of fibers in the image were calculated and exported for intergroup analysis. To eliminate errors arising from individual differences of rats, the functional test values before modeling of each rat were used as baseline values, and the data after modeling was normalized to the baseline values after 5 and 10 days of injection. The value was compared between different experimental groups for statistical significance. A two-way analysis of variance (2-way ANOVA) was performed using GraphPad Prism 9 software for the resting and systolic anal pressure, changes in EMG frequency and peak, DIL fluorescence intensity, and fiber proportions in different groups containing time and intervention factor groups, followed by the Benjamini test. The mean values were calculated for the 3 adjacent waveform groups selected for anal pressure and EMG, respectively, and *p* < 0.05 was considered statistically significant.

## 3. Results

### 3.1. Extraction and DIL Labeling of cSVF and tSVF

**Figure 1:**
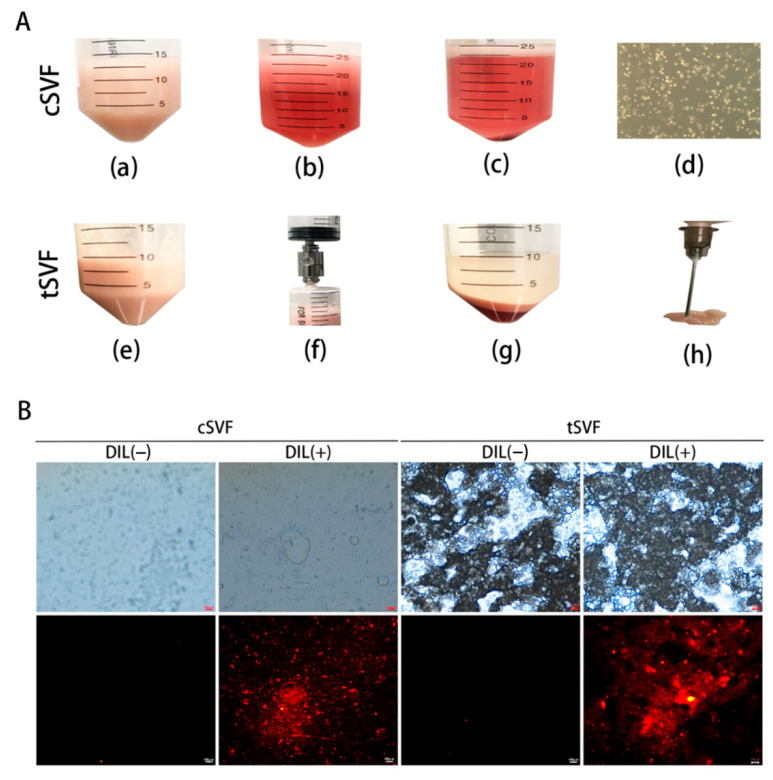
(**A**) Extraction process of cSVF (first row) and tSVF (second row). (**a**,**e**) Sheared fat particles; (**b**) adipose tissue after neutralization and digestion using DMEM complete medium; (**c**) centrifuged mixture; (**d**) cell morphology of cSVF in culture, after the first medium change; (**f**) fat passed through tSVF converter; (**g**) centrifuged emulsified celiac mixture; (**h**) 27 g needle injection of tSVF. (**B**) cSVF and tSVF with DIL markers. Left: cSVF on the left; Right: tSVF. First row: images under phase contrast microscope; Second row: images under fluorescent channel.

The images taken during the isolation of cSVF and tSVF were illustrated in Figure 1A. The cSVF obtained by enzymatic digestion method had high cell activity and relatively high cell volume (1 × 10^6^/mL). They resembled adipose stem cells in cell morphology in culture and had high proliferation. The tSVF obtained by mechanical emulsification method conformed to less than 15% of the original fat volume. The end product was injectable through a 27 g needle. The cell volume was no less than 6 × 10^4^/mL as determined by cell counting after sampling and enzymatic digestion.

CSVF and tSVF were labeled with DIL to allow in vivo tracing of these fractions after injection into animal models. The labeling effect was examined by microscopy. As shown in Figure 1B, only the cellular parts in cSVF and tSVF were labeled in red, but not the cell debris or lipid droplets.

### 3.2. Anal Pressure Results

**Figure 2:**
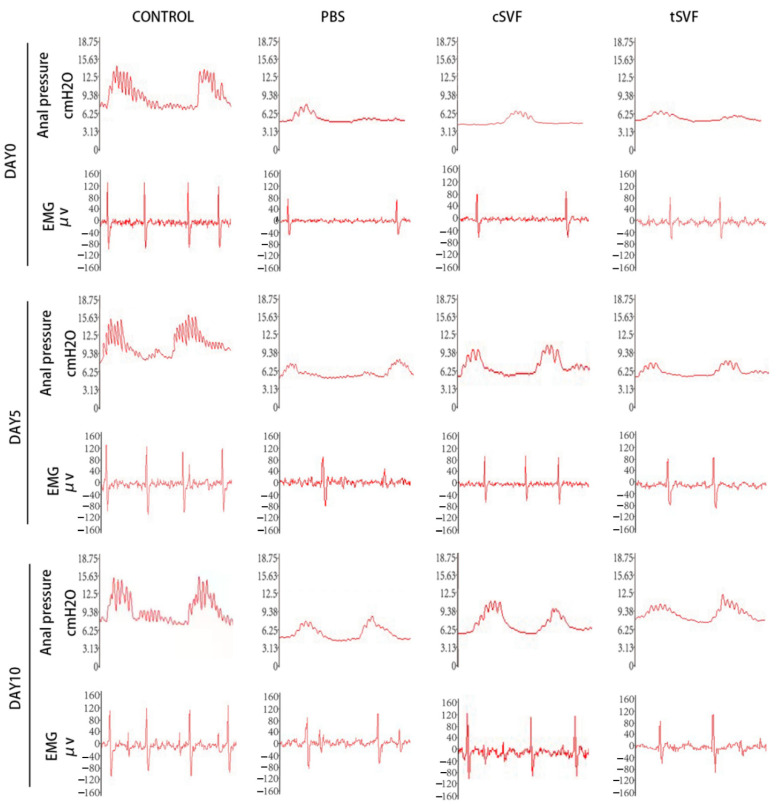
Waveforms of anal pressure and EMG in the control group, PBS group, cSVF group, and tSVF group were measured before treatment and at 5, 10 days after treatment. The waveform for anal pressure was recorded for 70 s, and the waveform for EMG was recorded for 160 ms.

**Figure 3:**
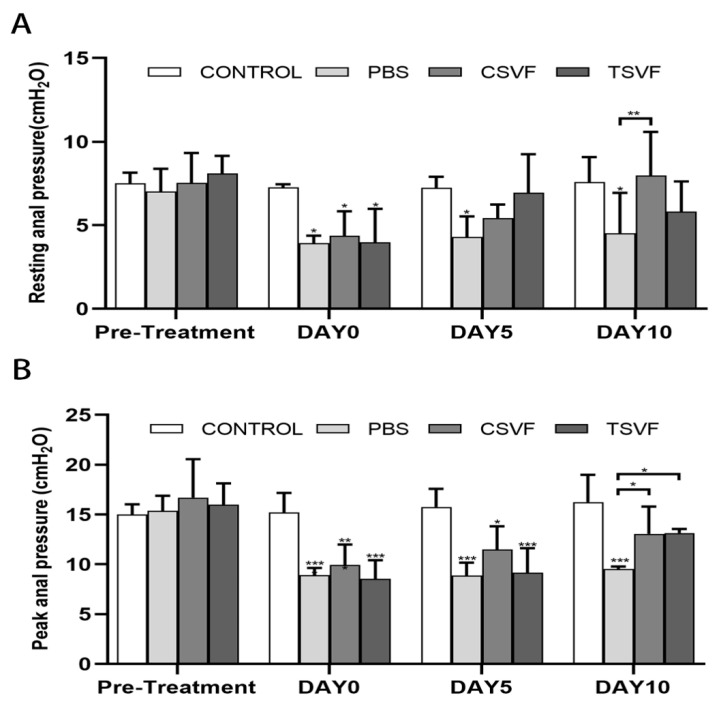
Statistical plots of the resting (**A**) and peak (**B**) values of anal pressure in the control group, the PBS group, the cSVF group, and the tSVF group before treatment and at 5, 10 days after treatment. All data was expressed as mean ± standard deviation, ‘*’, ‘**’, ‘***’ and represented statistical differences as *p* < 0.05, *p* < 0.01, *p* < 0.001, respectively. Those with horizontal lines represent statistical differences from the ends of the horizontal lines, and those without horizontal lines represent statistical differences compared with the control group.

Figure 2 showed the anal pressure recorded for a period of 70 s. The spontaneous rhythmic anal pressure waves were seen, with multiple systolic peaks for each waveform and inter-systolic intervals between adjacent anal pressure waves. The analysis of anal pressure was shown in Figure 3.

#### 3.2.1. Resting Anal Pressure Values

There was no statistical difference in the resting anal pressure values (7.01 ± 1.10, 7.53 ± 1.45, 7.22 ± 0.92, 8.09 ± 1.91) between the pre-injury control, PBS, cSVF, and tSVF groups. After modeling, the resting values of anal pressure in the PBS, cSVF, and tSVF groups (3.93 ± 0.35, 4.37 ± 1.27, and 3.98 ± 1.62) immediately before further treatment were significantly lower compared to the control group (7.27 ± 0.15, *p* < 0.05), indicating successful establishment of the injury model. On days 5 and 10, there was no significant trend of repair in resting values in the PBS group, suggesting difficulty in self recovery.

On day 5, the resting values in the tSVF group (6.95 ± 1.98) and the cSVF group (5.42 ± 0.66) were higher than the PBS group (4.28 ± 1.01), but the difference was not significant. On day 10, the resting value in the cSVF group (7.98 ± 2.26) continued to increase and was significantly higher than the PBS-treated group (4.51 ± 1.98, *p* < 0.05), indicating enhanced repair by cSVF. When comparing the two SVF groups with the control group, no statistical difference was found between the control group and the two SVF groups on both time points, indicating a reparative effect of both cSVF and tSVF. When comparing the cSVF group with the tSVF group, the values in the cSVF group were higher at both time points, but no significant difference was found, indicating a comparable effect of these two groups.

#### 3.2.2. Peak Anal Pressure

Similarly, as shown in Figure 2 and Figure 3, the peak systolic anal pressure was significantly lower in the PBS, cSVF and tSVF groups (8.93 ± 0.57, 9.94 ± 1.76, and 8.55 ± 1.52) compared with the control group (15.21 ± 1.60, *p* < 0.05) after modeling before further treatment. On day 5, the peak anal pressure in the cSVF group (11.47 ± 1.93) was higher compared to the PBS and tSVF groups (8.87 ± 1.06, 9.16 ± 2.13), but there was no statistical difference between these three groups, and they all have significantly lower peak anal pressure compared to the control group. On day 10, although the peak value in the tSVF group (13.14 ± 0.46) was still lower than that in the control group (16.25 ± 2.22), it was significantly better than that in the PBS group (9.53 ± 0.20), and the peak anal pressure in the cSVF group (13.07 ± 2.35) was also significantly higher than that in the PBS group. When the cSVF group was compared with the tSVF group, there was no significant difference at the two time points.

### 3.3. Electromyography Test Results

**Figure 4:**
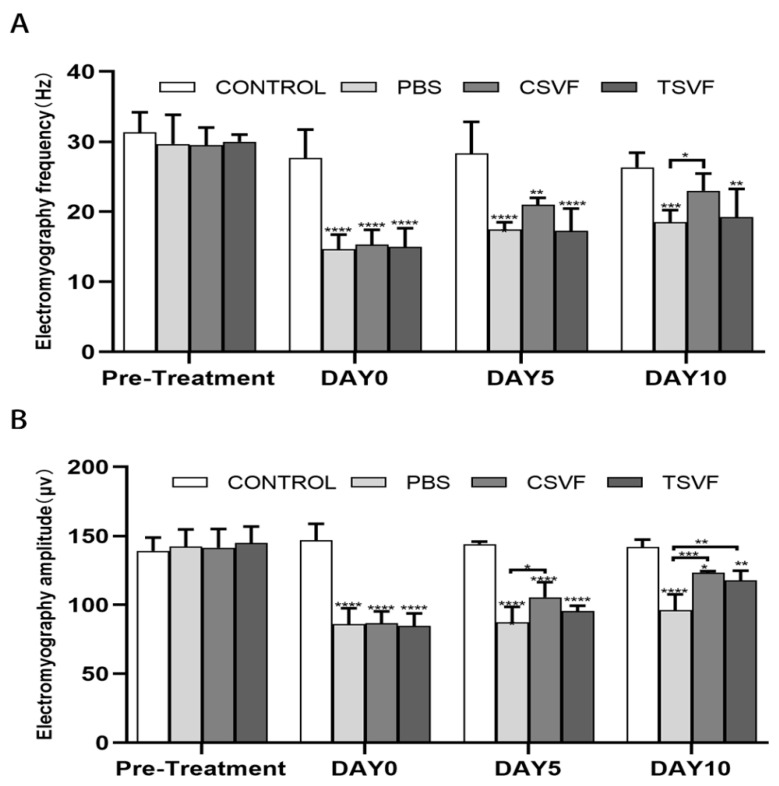
Statistical plots of EMG frequencies (**A**) and peaks (**B**) in the control group, the PBS group, the cSVF group, and the tSVF group before modeling and at 0, 5, and 10 days after cell treatment. All data was expressed as mean ± standard deviation. ‘*’, ‘**’, ‘***’, ‘****’ are represented as statistically different as *p* < 0.05, *p* < 0.01, *p* < 0.001, *p* < 0.0001, respectively. Those with horizontal lines represent statistical differences from the ends of the horizontal lines, and those without horizontal lines represent statistically differences compared to the control group.

Figure 2 shows the EMG recorded for 160 ms, which shows a clear motor unit action potential (MUAP) with stable waveform amplitude and frequency, and the analysis of electromyography frequencies and amplitudes was shown in Figure 4.

#### 3.3.1. Electromyographic Frequencies

The EMG frequencies of the groups before modeling (31.33 ± 2.35 in the control group, 29.66 ± 3.39 in the PBS, 29.50 ± 2.17 in the cSVF, and 30.00 ± 0.82 in the tSVF) were close with no significant differences. The frequencies of the PBS, cSVF, and tSVF groups (14.66 ± 1.69, 15.33 ± 1.69, 15.00 ± 2.16) were significantly decreased immediately after modeling compared with the control group (27.66 ± 3.29), confirming the establishment of the modeling. On day 5, the cSVF group scored higher (21.00 ± 0.81) than the PBS group (17.50 ± 0.86) and the tSVF group (17.25 ± 2.77), but no statistical difference was found among these three (*p* > 0.05), which were all significantly lower than the control group. On day 10, the frequency of the cSVF group (23.00 ± 2.21) was significantly higher than that of the PBS group (18.50 ± 1.50), while it was close to that of the control group, and there was no statistically significant difference between them, indicating complete recovery of EMG frequency. In contrast, tSVF group (19.25 ± 3.49) did not present significant improvement over the PBS group. When comparing the cSVF group with the tSVF group, the values of cSVF group were higher with no significant difference at both time points.

#### 3.3.2. EMG Amplitude

The EMG amplitudes were basically the same in each group before modeling (control: 139.17 ± 7.83, PBS: 142.27 ± 10.09, cSVF: 141.51 ± 11.69, tSVF: 144.93 ± 9.74), with no significant differences. After modeling, the amplitude of the PBS, cSVF, and tSVF groups (85.98 ± 9.41, 86.69 ± 6.94, 84.63 ± 7.48) decreased significantly compared with the control group (146.89 ± 9.69). On day 5, the EMG amplitude of cSVF group (105.52 ± 8.98) was significantly higher (*p* < 0.05) compared to the PBS group (87.39 ± 9.66), while the EMG amplitude of the tSVF group (95.57 ± 3.30) was slightly higher than the PBS group with no statistical difference. On day 10, the cSVF group (123.41 ± 1.88) increased significantly compared to the PBS group (96.10 ± 10.09) and approached the value in the control group (141.93 ± 4.36), still with significant difference. The EMG amplitude of the tSVF group (117.83 ± 6.03) was also statistically higher compared to the PBS group. When comparing the two SVF groups, the values in the cSVF group were higher with no significant difference at both time points.

### 3.4. Surface Healing of the Injury Site

**Figure 5:**
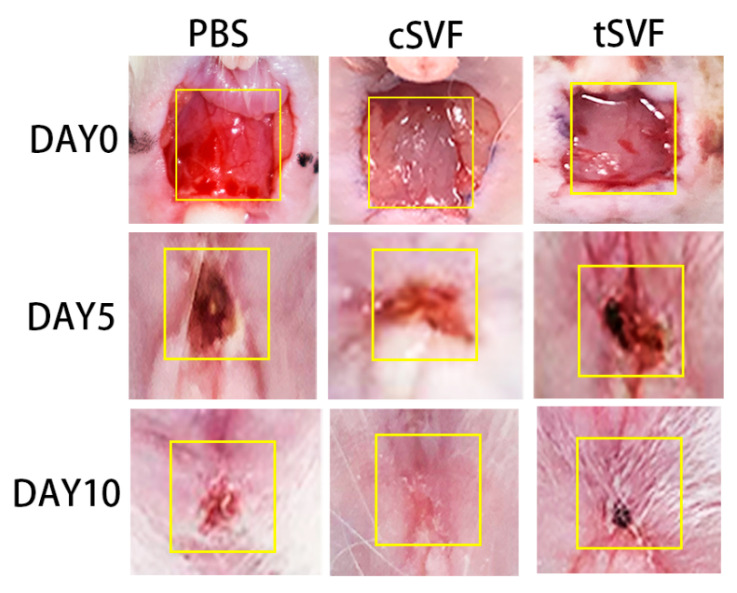
Surface healing of the injury site at 0, 5, and 10 days after receiving treatment in the PBS, cSVF, and tSVF groups. Yellow boxes represent injury sites.

Figure 5 illustrates the appearance of the injury site for each group at 5 and 10 days of treatment. Immediately after modeling, a large defect was seen between the anus and urethra in all rats. On day 5, the wounds in the PBS, cSVF, and tSVF groups became significantly smaller compared to day 0, and the SVF treatment group had smaller wounds compared to the PBS group. On the 10th day after treatment, the PBS group still had incomplete healing at the wound site, and the wound in the tSVF group is even smaller but still visible. The cSVF group healed best and was close to complete healing.

### 3.5. Masson Trichrome Stain

**Figure 6:**
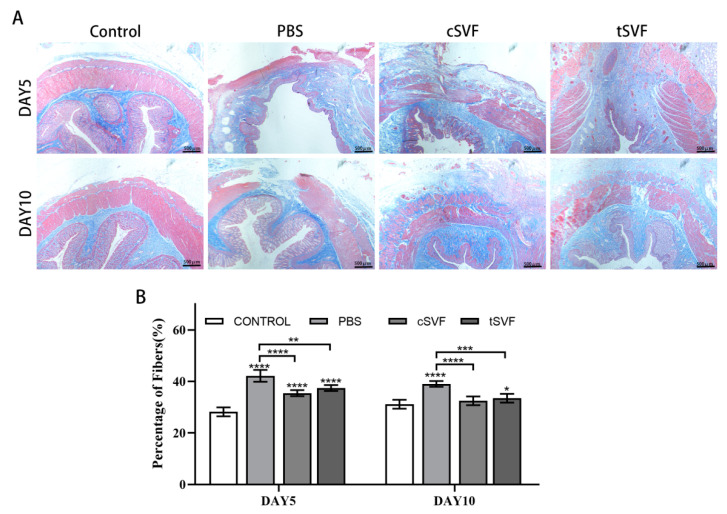
Masson trichrome staining plots (**A**) and fiber scale plots (**B**) of the control group, PBS group, cSVF group and tSVF group at 5 and 10 days after treatment. ‘*’, ‘**’, ‘***’, ‘****’ represent statistically different as *p* < 0.05, *p* < 0.01, *p* < 0.001, *p* < 0.0001, respectively. Those with horizontal lines represent statistical differences from the ends of the horizontal lines, and those without horizontal lines are statistically different compared with the control group.

Masson trichrome stains were performed on the retained anal sphincter specimens (Figure 6A). Large defects were still present in the PBS group at day 5, which were larger compared to those in the cSVF and tSVF groups. Defects were still visible in the cSVF group at day 5, but significantly more muscle was formed compared to the PBS group. On day 5, the tSVF group had larger defects than the cSVF group. On day 10, most of the gaps in the PBS group had been filled with new muscle and fibers, but the defects were still larger compared to the cSVF and tSVF groups. The area of defect in the cSVF group was almost healed, while a smaller defect can be detected in the tSVF group.

Quantitative fiber analysis of the specimens was performed to evaluate the extent of fibrosis in the injured area (Figure 6B). The results showed that on day 5, both SVF groups (cSVF: 35.46 ± 0.95, tSVF: 37.46 ± 0.96) had a higher proportion of fibers than the control group (28.18 ± 1.81), and was significantly lower than the PBS groups (42.41 ± 1.87), indicating an anti-fibrotic effect. The proportion of fibers was slightly higher in the tSVF group than in the cSVF group, but there was no statistical difference (*p* > 0.05). On day 10, the fiber proportion in the cSVF and tSVF groups (cSVF: 32.50 ± 1.39, tSVF: 33.46 ± 1.29) was significantly lower than that in the PBS group (39.06 ± 0.90). The fibers in the cSVF group recovered to the level of the control group (31.15 ± 1.42) and there was no significant difference between the two groups. For the tSVF group, although the fiber proportion further decreased at day 10 compared to day 5, it was still significantly higher than that of the control group.

### 3.6. Immunofluorescence Staining of cSVF and tSVF

**Figure 7:**
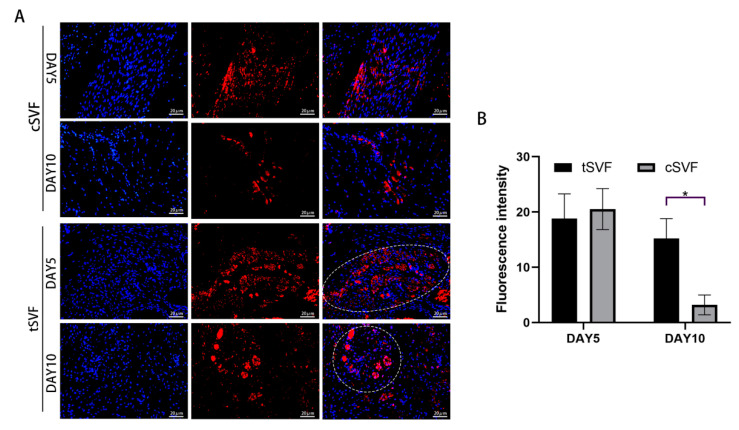
Fluorescence distribution of the cSVF and tSVF groups after 5 and 10 days of treatment. (**A**) shows the immunofluorescence image of DIL expression, with white circles indicating the area of concentrated fluorescence distribution; (**B**) shows the analysis of fluorescence intensity in the cSVF and tSVF group. ‘*’ indicates a statistical difference of *p* < 0.05.

We performed immunofluorescence staining to understand whether cSVF and tSVF survive, proliferate, and differentiate towards target cells in vivo. Figure 7 shows the distribution and survival of cSVF and tSVF in vivo after injection. The results showed that surviving cells could be detected in both SVFs at 5 and 10 days after injection, while the fluorescence intensity of the cSVF group continued to diminish at day 10 and was significantly lower than that of the tSVF group. This may be due to the fact that tSVF tended to aggregate together so it showed an uneven distribution, while cSVF showed a scattered distribution throughout the injury area.

**Figure 8:**
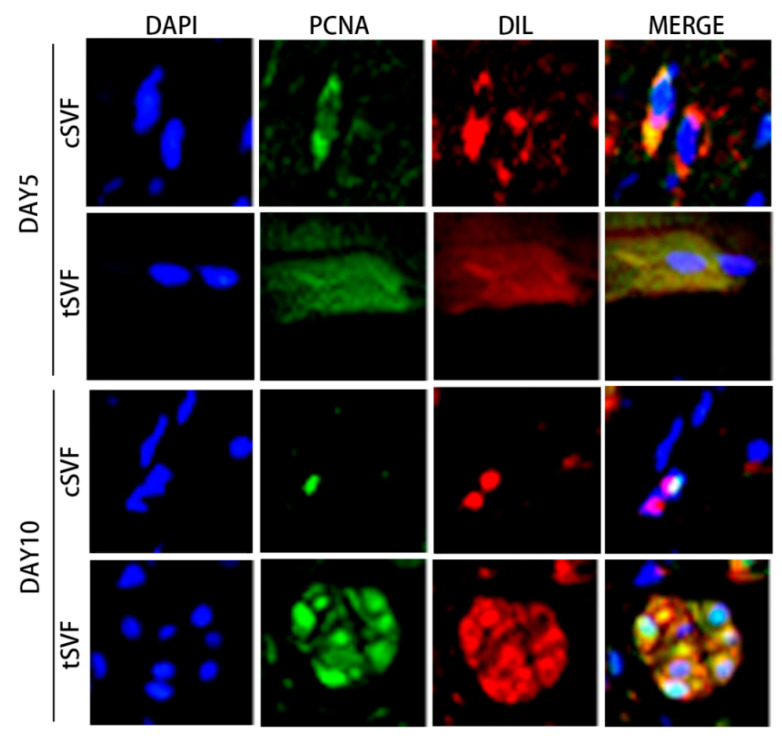
Cells co-expressed DIL and PCNA at day 5 and day 10 post injection in the cSVF and tSVF group. PCNA: proliferating cell nuclear antigen.

Cells in both cSVF and tSVF were found to be in a proliferative state, as represented by the expression of PCNA, a proliferation marker, on both day 5 and day 10 (Figure 8).

**Figure 9:**
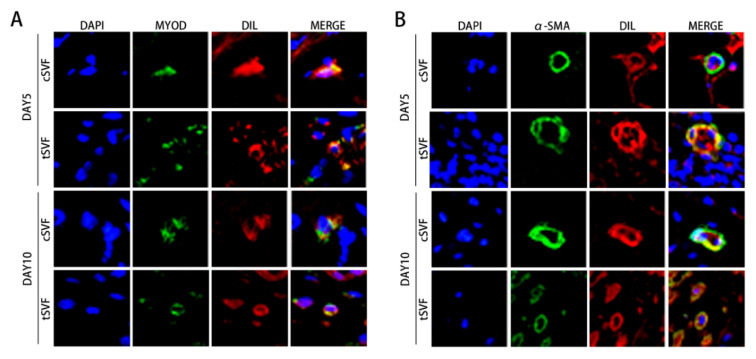
CSVF and tSVF differentiated into myosatellite cells and smooth muscle cells in vivo. Some Dil-labeled cells expressing MYOD (**A**) or α-SMA (**B**) were detected in the cSVF and tSVF groups on days 5 and 10 after treatment. MYOD: myogenic differentiation factor D, α-SMA: α smooth muscle actin.

We also examined the generation of injected cells towards myosatellite cells, represented by MYOD expression, and towards smooth muscle cells, represented by α-SMA expression. The results showed that both cSVF and tSVF group had some DIL positive cells expressing MYOD or α-SMA at both day 5 and day 10 (Figure 9), suggesting that some cells in cSVF and tSVF have differentiated into target cells (smooth muscle cells) or their precursor cells (myosatellite cells).

## 4. Discussion

ADSC is a type of adult stem cells. They can differentiate into adipocytes, chondrocytes, osteoblasts, myocytes, hepatocytes, and other cells [16,40,41,42,43,44,45,46,47], and can secrete some cytokines and extracellular matrix (ECM) [48,49]. ADSC are similar to bone marrow mesenchymal stem cells (MSC) [50,51], therefore they are considered to be adipose tissue derived MSCs, which can be isolated from various tissue sources [52] and possess similar characteristics with some differences in proteomics and differentiation capacity [53,54,55,56]. In contrast to ADSC for which the preparation requires in vitro culture amplification, SVF can be prepared easily and rapidly without in vitro expansion, which facilitates clinical application. TSVF has advantages (Table 1) over cSVF in that its preparation does not involve enzymatic digestion. In clinical applications, it is generally agreed that the introduction of exogenous substances should be avoided as much as possible, and studies have shown that the inclusion of collagenase in injections may cause serious side effects in the body, such as skin ulcers, nerve damage, tendon damage, and allergic reactions [38]. Therefore, the clinical application of the enzymatic method is somewhat limited. In contrast, the mechanical separation method has great potential for application without the introduction of exogenous digestive enzymes and low preparation cost.

The impact of different tissue source on the activity, composition, and post-preparation yield of SVF has been investigated. SVF in animal experiments is usually obtained from the groin [57], epididymis [32], etc. In clinical studies, fat is often obtained by liposuction or manual liposuction [58,59,60,61]. Sinno et al. [62] showed no significant differences in the activity of SVF prepared from different sites of fat. Tsekouras et al. [63] isolated SVF from liposuction of human abdomen, waist, and inner and outer thighs, respectively, and found that different sites have minimal impact on the final product activity, but the largest number of SVF cells was collected from the inner thigh. In our study, we used visceral fat but not subcutaneous fat, since we need high volume of SVF for the experiment, and rats have more visceral fat than subcutaneous fat.

Preparation methods may also lead to differences in the activity, composition, and post-preparation yield of SVF. In van Dongen’s study, cell yields by enzymatic digestion were typically in the range of 0.19–11.7 × 10^5^ cells per 1 mL of fat before treatment, while cell yields by non-enzymatic digestion were typically in the range of 1.8–22.6 × 10^5^ in 1 mL of end product [35]. Chaput B et al. [64] compared the yield and characteristics of SVF obtained by enzymatic digestion and mechanical separation methods, and found that cSVF had greater yields of cells than tSVF, but cSVF and tSVF were comparable in terms of cell phenotype, differentiation potential, and immunosuppressive properties. In our study, to enable a as fair as possible comparison between the two SVF treatments, we use the same initial volume of fat to prepare cSVF and tSVF, and the end product was quantified to the same volume, usually with a larger volume of tSVF, and the cSVF was supplemented with PBS to the same volume as tSVF, therefore we can ensure that the amount of cSVF and tSVF for each injection was originally isolated from the same initial volume of fat.

Factors that affect the activity of cSVF during preparation include the type and concentration of digestive enzymes, as well as the digestion time. The digestive enzymes used in the literature include one or a combination of the following: collagenase, dispase, trypsin, or related enzymes. The digestion time varies from 30 min to 60 min [37,65]. In our study, the digestion time and collagenase concentration were optimized to 0.075% type I collagenase for 30 min, which is consistent with most of the methods reported in the literature [32]. In our practice, insufficient digestion time decreases the amount of cells obtained, while prolonged digestion time increases the number of dead cells. The tSVF preparation requires processing through rotation [66] or SVF converters of different pore sizes, luer joints or other fully automated separation equipment, which probably has great impact on cell activity. The study by Rik Osinga et al. [67] showed that the macroscopic structure of the adipose tissue changed during the homogenization in the converter as the number of pushes in the converter increased, but there were no significant changes in microstructure as well as cell content, activity, and cell ratio. However, there were significant differences in the separation rate, clonality, and viability of SVF obtained from different donor fats. In our study, we reduced this error due to individual variations by mixing all collected fat and dividing equally into two.

The anal sphincter complex is the structural basis for the regulation of anal function, with the internal anal sphincter primarily maintaining resting tone, while the external anal sphincter can be adjusted according to self-will. In this way, the functional status of the internal and external anal sphincter is reflected by testing the resting and contractile anal tone. We found that on day 5, there was no statistical difference in either anal pressure resting tone, peak anal pressure contraction, or EMG frequencies between the tSVF, cSVF groups and the PBS group, but the peak EMG in the cSVF group showed a significant increase compared to the PBS group. On day 10, the EMG and anal pressure indices in the cSVF group showed a significant increase compared with the PBS group. The tSVF group also showed significant improvements in peak anal pressure and peak EMG, and the resting anal pressure values on day 5 and the peak anal pressure values on day 10 were slightly higher with no significance in the tSVF group than in the cSVF group. This indicates that cSVF had slightly higher improvement than tSVF in restoring anal pressure and EMG than tSVF, yet with no significant difference. We conjecture that the main reason for this difference is the different mechanism by which cSVF and tSVF act in vivo, because we found that cSVF showed a scattered distribution in the damaged area, while tSVF showed an aggregated distribution, which may limit the exertion of its therapeutic effect. In addition, the observation time may matter, as a longer time point may result in a different outcome.

The mechanisms by which SVF exerts its therapeutic effects are still unclear. Studies have shown that each cell in SVF can act independently or synergistically, including immunomodulation, angiogenesis, and intercellular synergism [32,66,68,69,70,71]. Dai et al. [72] showed that SVF-secreted exosomes play an important role in wound repair. Zhou et al. [32] found that the expression of alpha (TNF-α) decreased and the expression of interleukin 10 (IL-10) increased after SVF treatment, while the neutrophil content decreased and the M2 type macrophages increased. ADSC in SVF produce IL-1 receptor antagonists and promote polarization and expression of the histoprotective protein tumor necrosis factor-stimulated gene (TSG-6) in nonpolarized macrophages and mature dendritic cells toward an anti-inflammatory and phagocytic phenotype. In contrast, in a study by Dong, ZQ et al. [73], it was shown that macrophages, which account for 20% of SVF (70% M2 type), have the effect of reducing inflammatory response and promoting angiogenesis, and there is an increase in M2 macrophages after SVF treatment. SVF can also secrete growth factors, neurotrophic factors, and adipokines. These cytokines can promote neovascularization. Çağatay Öner et al. [57] showed that SVF can also attenuate tissue fibrosis. Guerrero et al. [74] found that T-cadherin-positive cells in SVF was found to promote the osteogenesis of SVF cells in vivo and strongly support the formation of vascular networks through the interaction of vascular endothelial growth factor and adiponectin.

Few studies have investigated the differentiation to target cells of SVF in vivo. The internal anal sphincter and external anal sphincter are constructed by smooth and skeletal muscles, respectively [39]. After muscle injury, neighboring myosatellite cells first migrate to the lesion site and enhance mitotic activity [75,76]. In our study, we found that injected SVF was still alive and in a proliferative state at 5 and 10 days after DRBinjection, and some cells from cSVF and tSVF differentiated into myosatellite cells and smooth muscle cells, suggesting that SVF may promote muscle repair through differentiation to mature target cells in addition to its anti-inflammatory and paracrine effects.

In this study, we also observed a phenomenon that tSVF did not migrate significantly in vivo over time and remained aggregated, while cSVF, on the other hand, migrated distantly leaving a small number of cells remaining in the original injection location over time. We suspect this is mainly due to the fact that the fibrous matrix in the tSVF limited the ability of tSVF to migrate, while cSVF is not affected as the fibrous component of cSVF has already been digested by enzymes. Most of the cells in tSVF within the scaffold were in a proliferative and differentiated state, while studies by Derek A. Banyard et al. [77] showed an increase in their pluripotency after mechanical compression. Microenvironment where stem cell harbors can offer support and impose influence on stem cells [78], however, in this study the functional repair of the anal sphincter by tSVF remained slightly suboptimal as compared to cSVF in the observation period. We suspect that this is probably due to the fact that we injected cells at the muscle layer adjacent to the injury site, and the cells in the tSVF cannot migrate out as free as cSVF affected the tSVF to exert their reparative effect directly at the injury site. Future strategies to improve the distribution of tSVF may help to promote their effect to repair ASI.

SVF treatment was shown to be safe in most of the reports in the literature. However, Heidi Ledford [79] reported three patients with macular degeneration of the eye that resulted in eye blindness after cSVF treatment, and Suzanne E. L.’s study [80] found a severe inflammatory response, including lymphocytic infiltration, angiogenesis, and disc damage, after treatment of degenerated goat intervertebral disc models. Adverse events associated with tSVF have not been reported. In our study, though we have not directly assessed the markers for tumorgenicity in our experiment, we have not observed the formation of tumors during the treatment, nor any other signs of complications or deaths in the rats.

We acknowledge that the present study has preliminarily explored the mechanisms involved in the therapeutic effects of cSVF and tSVF in vivo, which showed that cSVF and tSVF could proliferate in vivo to regenerate the damaged tissue, and they could differentiate into target cells, including myosatellite cells and smooth muscle cells, but the factors regulating these cells to differentiate to the target cells are still unclear. Future studies to explore the molecular mechanism to drive the differentiation of SVF will be desired, which may provide clues to enhance the effect of SVF on ASI repair. In addition, the way to inject tSVF may need further optimization, to facilitate a more even distribution or migration of tSVF in the injury area in vivo, and to provide guidelines for future clinical application.

## 5. Conclusions

Both cSVF and tSVF showed reparative effects on ASI. While the effect of cSVF is slighter higher with no significant difference, cSVF and tSVF were still alive and in a proliferative state after 10 days of in vivo injection, while some cells in both cSVF and tSVF could differentiate to myosatellite cells and smooth muscle cells.

## Figures and Tables

**Table 1:** Comparison of the advantages and disadvantages of cSVF and tSVF.

	cSVF	tSVF
Composition	Cellular components (ADSC, pericytes, endothelial cells, etc.)	Cellular components (ADSC, pericytes, endothelial cells, etc.), ECM, cytokines
Digestive enzymes	Require	Not required
Time consumption	Long	Short
Security	Have side effects	Not found yet
Price	Expensive	Cheap

## Data Availability

Data available on request from the corresponding author.
